# Telemonitoring in Long-COVID Patients—Preliminary Findings

**DOI:** 10.3390/ijerph19095268

**Published:** 2022-04-26

**Authors:** Anna Romaszko-Wojtowicz, Stanisław Maksymowicz, Andrzej Jarynowski, Łukasz Jaśkiewicz, Łukasz Czekaj, Anna Doboszyńska

**Affiliations:** 1 Department of Pulmonology, School of Public Health, Collegium Medicum of the University of Warmia and Mazury, ul. Jagiellońska 78, 10-357 Olsztyn, Poland; anna.romaszko@uwm.edu.pl (A.R.-W.); anna.doboszynska@wp.pl (A.D.); 2 Department of Psychology and Sociology of Health and Public Health, School of Public Health, Collegium Medicum of the University of Warmia and Mazury, al. Warszawska 30, 10-082 Olsztyn, Poland; 3 Aidmed Sp. z o.o., ul. Uphagena 27, 80-237 Gdańsk, Poland; ajarynowski@aidmed.ai (A.J.); lukasz.czekaj@aidlab.com (Ł.C.); 4 Department of Human Physiology and Pathophysiology, School of Medicine, Collegium Medicum of the University of Warmia and Mazury, al. Warszawska 30, 10-082 Olsztyn, Poland; lukasz.jaskiewicz@uwm.edu.pl

**Keywords:** telemedicine, long-COVID, cardiological and respiratory disorders

## Abstract

The COVID-19 pandemic has revealed the high usefulness of telemedicine. To date, no uniform recommendations or diagnostic protocols for long-COVID patients have been developed. This article presents the preliminary results of the examination of patients after SARS-CoV-2 infection who were provided with medical telemonitoring devices in order to oversee their pulmonological and cardiological health. Three cases have been analyzed. Each patient underwent a 10-day registration of basic vital signs, in three 15-min sessions daily: RR (respiratory rate), ECG (electrocardiogram), HR (pulse), SPO_2_ (saturation), body temperature and cough. Rule methods and machine learning were employed to automatically detect events. As a result, serious disorders of all the three patients were detected: cardiological and respiratory disorders that required extended diagnostics. Furthermore, average values of the selected parameters (RR, HR, SPO_2_) were calculated for every patient, including an indication of how often they exceeded the alarm thresholds. In conclusion, monitoring parameters in patients using telemedicine, especially in a time of limited access to the healthcare system, is a valuable clinical instrument. It enables medical professionals to recognize conditions which may endanger a patient’s health or life. Telemedicine provides a reliable assessment of a patient’s health status made over a distance, which can alleviate a patient’s stress caused by long-COVID syndrome. Telemedicine allows identification of disorders and performing further diagnosis, which is possible owing to the implementation of advanced analysis. Telemedicine, however, requires flexibility and the engagement of a multidisciplinary team, who will respond to patients’ problems on an ongoing basis.

## 1. Introduction

COVID-19 (Coronavirus Disease 2019) is a highly infectious respiratory system disease caused by a new coronavirus called SARS-CoV-2 [1]. COVID-19 infection is a heterogenic disease displaying a wide array of symptoms, from mild ones, such as dry cough and headache, to a full-blown respiratory failure, kidney failure and sudden cardiac arrest. The mortality rate due to COVID-19 is considerable. Amstrong et al. have performed a meta-analysis of the data from 52 studies in different countries, which showed that between 42.2% and 98.8% of COVID-infected patients need to be hospitalized, and the average mortality rate among them is 35.5% [2]. Patients with medical history, cardiovascular diseases or a tobacco-smoking habit are exposed to the risk of a more severe course of the illness, with respiratory insufficiency, renal insufficiency, cardiac arrhythmias or shock [3,4,5]. The problem of high mortality due to COVID-19 is also visible in the general statistics of the World Health Organization. According to WHO data for 29 March 2022, there have been 481,756,671 confirmed cases of COVID-19 globally, including 6,127,981 deaths, giving a 1.27 percent mortality rate [6].

During the COVID-19 pandemic, remote communication has found application in many areas of medicine. Telemedicine has many different advantages and disadvantages that vary depending on the method of communication (table comparison can be found in the Supplementary Materials). In the present study, thanks to the use of remote patient registration methods, despite the lack of the possibility of physical examination, we were able to monitor patients’ health status in real time. Thus, in an objective manner, the physician is able to verify at least some of the symptoms reported by the patient thanks to such methods as: the measurement of saturation, registration of the respiratory rate or ECG recording. 

A separate group of patients are patients with the so-called long-COVID syndrome. After COVID-19 infection, patients need to be monitored as some may have persistent symptoms, such as fatigue, cough, dyspnea, reduced physical performance after a severe course of the SARS-CoV-2 infection, etc. [7]. It is important to pay attention to patients with symptoms indicating a prolonged course of the illness (long-COVID) [8]. Depending on the duration of ailments after recovering from a SARS-CoV-2 infection, two groups can be distinguished: acute, where such symptoms last for over 3 weeks but no longer than 12 weeks; and chronic, where symptoms persist for over 12 weeks [9]. The term ‘long COVID’, which refers to the latter duration of symptoms, was first used by Pere on social media in order to underline the persistence of COVID-19 symptoms after a few weeks or even months since the initial infection with the SARS-CoV-2 virus [10]. It is worth noting that the adversely affected well-being of patients after COVID-19 can also result from the psychosomatic pathogenesis of the disease [11], hence the importance of the psychological monitoring of such patients.

Telehealth is the provision of healthcare by healthcare professionals via information technology and electronic communication channels [12]. Telemedicine, as a subcategory of the health service, can take the following forms: online consultation, telemonitoring or video chats [13]. According to the report by the Centers for Diseases Control and Prevention, issued in October 2020, the number of telehealth visits in the United States in the first quarter of 2020 was 50% higher than in the same period of time in 2019 [14]. This evident change in patient diagnosis and treatment has encouraged the expansion of telemedicine possibilities. In order to satisfy patients’ needs and expectations, numerous commercial firms have joined the efforts to expand a range of diagnostic applications available through telemedicine channels. 

This preliminary report aims to present the results of a study which covered patients post-SARS-CoV-2 infection. The article contains a description of three cases of patients who were detected with the Aidmed One system to suffer from serious health disorders that necessitated further diagnosis. It needs to be emphasized that this is an ongoing study, and the intention of the authors is to give a preliminary presentation of diagnostic possibilities created by the use of new technologies in telemedicine rather than to present the results of the mentioned study. Thus, the main objective of this article is to share our practical experience and discuss strengths, opportunities, threats and weaknesses in technological (i.e., usability), medical (i.e., clinical applicability) and social (i.e., inclusiveness) aspects of remote diagnostics of long-COVID patients.

The following work is divided into the following sections: Section 2 describes the study design and data collection and the measurement methods in detail; Section 3 contains the separate measurement results for each patient, with a detailed description of the patient’s medical history, the obtained data on their condition and the intervention undertaken, resulting from the obtained data; Section 4 is a discussion of the obtained results against the background of other works and problems; and finally, Section 5 contains the conclusions of our study and shows the limitations to which it is subject.

## 2. Materials and Methods

### 2.1. Study Design and Data Collection

The study consists remote recording and the assessment of vital functions using telemedicine instruments. A certified, class IIa medical device called Aidmed, used in this study, is a modern system applied to measure several of a patient’s physiological functions. The system is composed of a certified device (brand name: *Aidmed One Recorder*), a mobile application Aidmed Health available on iOS and Android systems, and a telemedicine system named Aidmed Cloud [15]. 

The following persons are disqualified: underage, incapacitated, incarcerated, pregnant women, persons with cognitive dysfunctions, those unable to perform the minimum set of clinical measurements (e.g., because of obesity or skin diseases) and those with an implanted cardiac pacemaker. During the qualification, medical history and physical examination were performed for every patient to create their simplified health profile. Only patients who have gone through a COVID-19 infection confirmed by a smear test can be qualified for the study.

The patients gave their consent to participate in the study, thereby being obliged to take measurements at least three times a day for 15 min, at the same time, for at least 10 days. Every patient can also take measurements at night in case of OSA (Obstructive Sleep Apnea) and perform a functional exercise test. During the qualification process and the study itself, a patient completes several questionnaires (described in the section ‘Measurements’). To date (as of 12 September 2021), 77 patients have been qualified to partake in the study, conducted at the Department of Pulmonology in Olsztyn. The study is now in progress. 

The above work presents the measurement results for three selected patients in accordance with the purposeful sampling method [16]. The work is therefore of a qualitative nature, presenting preliminary findings. Upon the completion of the study, a paper based on the entire study sample will be submitted.

The experiment has been issued an approval by the Bioethics Commission at the University of Warmia and Mazury in Olsztyn, the approval decision no 17/2021. The entire non-commercial research, conducted in several centers, is funded by the Medical Research Agency (grant no 2020/ABM/COVI19/0082).

### 2.2. Measurments

The measurements are registered by the Aidmed One recorder, which is fitted on an elastic belt wrapped around the chest at the height of the xiphoid process of the sternum. A single-channel ECG signal is collected by silicone electrodes mounted on a belt worn on the chest. The respiratory rhythm is determined according to the changes in the electrical impedance of the chest that occur when the chest changes its shape during respiration. The recorder is coupled with a nasal cannula, which is a differential air-pressure sensor that enables the system to provide additional evaluation of the rhythm and depth of breaths. Furthermore, using a contact-temperature sensor, the patient’s skin temperature is taken. The Aidmed One recorder also features an in-built microphone, useful to detect coughing or snoring. Another component of the system is a wireless fingertip pulse oximeter coupled with the main application for the determination of blood saturation. The Aidmed One device records the ECG signal, which allows early detection of cardiac arrhythmias; for example, supraventricular tachycardia or ventricular pre-excitations. As early as during the qualification process, patients are informed about any worrying test results that require urgent medical intervention. 

Using an application in one’s mobile telephone or by contacting a telephone consultant, patients provide the following information: (1) socio-demographic data; (2) data concerning the quality of life WHOQOL-BREF and longitudinal EQ-5D-5L; (3) longitudinal symptom questionnaires; (4) assessment of (e-) health literacy according to the questionnaires HLS-EU-Q16 and Pl-eHEALS; (5) evaluation of loneliness, isolation, stress and anxiety; (6) perceived usability of the technology according to SUS.

The data collected during the study are transmitted via Bluetooth technology to a mobile telephone, and then to a server, the access to which is given to the physician conducting the study. 

In order to assist the doctor monitoring the patient remotely, a preliminary automated analysis of signals was employed. The following machine learning algorithms have been implemented: (1) apnea and shallow breath using air flow and saturation data; (2) ectopic (supraventricular) disorders using ECG (electrocardiogram) readings. In addition, rule classifiers applied to HR (heart rate), RR (respiratory rate) and SpO_2_, based on cut-off thresholds which are determined by the physician for every patient (selected threshold values are given in the tables below) enable the preselection of selected signal fragments that call for further intervention. 

The detectors implemented to detect apnea, dyspnea and desaturation were developed in line with the AASM guidelines [17]. Rule detectors of the above events were designed, based on the course of the envelopes of relevant signals. Each envelope was determined from multiple peak-to-valley statistics. Initially, maximum and minimum values of the signal were filtered in order to improve the quality of the envelope being determined. The parameters of the filters and detectors were adjusted based on training sets. Arrhythmia classifiers were developed according to deep learning methods trained on publicly available sets of data. Classification was achieved on the basis of single cardiac cycles broken into the following classes: non-ectopic, ectopic–ventricular, ectopic–supraventricular, fusion, artefact. The classification and approach to validation are in accordance with the guidelines [American National Standard for Ambulatory Electrocardiographs (ANSI/AAMI EC38:1998)]. The classifier was validated based on the set (MIT DB: The Massachusetts Institute of Technology—Beth Israel Hospital Arrhythmia Database (48 records, 30 min each)).

## 3. Results

### 3.1. Patient 1

The first patient is a 46-year-old man, previously not treated for any chronic diseases. On 17 February 2021, the patient was confirmed to be infected with the SARS-CoV-2 virus. Education: secondary. Hospitalized earlier due to a spinal disease and COVID-19. He was a non-smoker for over 10 years. 

**Figure 1:**
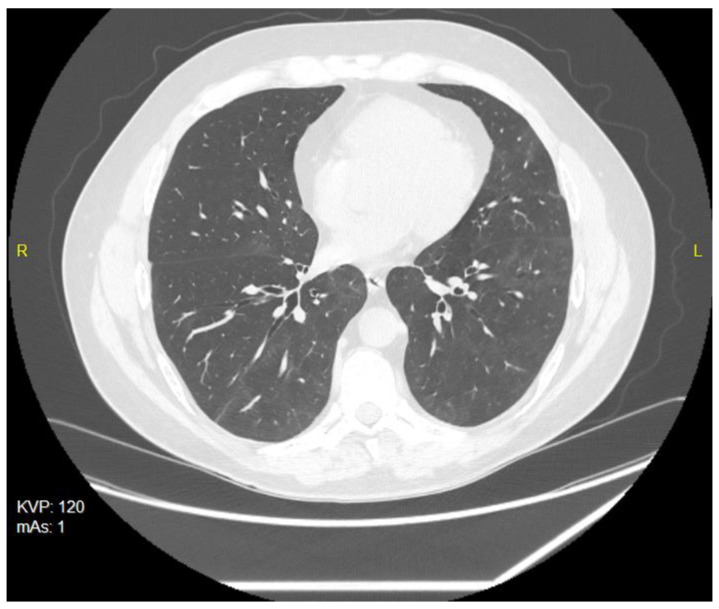
CT scan of the chest of 20 February 2021—small, bilateral infiltrates of the grain-glass opacity type.

Due to the generally poor health condition, breathlessness, cough and fatigue, the patient needed to be hospitalized in a dedicated ward from 17 February 2021 to 1 March 2021. The chest X-ray performed during his hospital stay revealed consolidations of the lung parenchyma, while the CT (computer tomography) of the chest performed on 20 February 2021 showed that 55–60% of the lung parenchyma was invaded. The following control chest CT on 21 May 2021demonstrated an almost complete regression of inflammatory changes. Persistent changes included small, transient areas of ground-glass opacity, as well as linear fibrosis with traction bronchiectasis and sings of disturbed pulmonary architecture in the lower fields of both lungs and on segments of the lingula (Figure 1). 

During the qualification for the study, the patient complained of slight dyspnea on exertion (mMRC 2), arterial blood pressure 171/107 mmHg and HR 97/min. The monitoring with Aidmed One lasted from 20 August 2021 until 3 September 2021and comprised 15 h 49 min of measurement taking. During that time, 191 events of elevated pulse (over 100 beats/minute) were recorded, with no arrhythmias, as the ECG readings showed no ventricular and supraventricular extrasystoles (Table 1). 

The patient was recommended further cardiological diagnosis. 

The results of the patient’s questionnaires are as follows: the quality of sleep was assessed as average. The patient does not pay attention to healthy eating habits. He does regular physical exercise. He is not allergic to anything, does not take dietary supplements or herbs and occasionally consumes alcohol. On the EQ_5D_5L life-quality assessment scale, the patient did not indicate any limitations and evaluated his health as the best possible (99/100 points). In the WHOQOL_Bref questionnaire, the patient did not indicate any physical, mental or social limitations in everyday functioning, and evaluated the quality of his life as good. The patient did not mark any problems on a scale measuring the sense of loneliness (The UCLA LS). He experienced an average stress level. He declares good knowledge of his health and high e-health competence. He had no difficulty operating Aidmed. He did not manifest any burden caused by the pandemic and adheres to the restrictions.

The increased heartbeat frequency could have been caused by several reasons, such as metabolic, hematological or cardiological disturbances, the latter represented by conduction abnormalities inducing extra ventricular or supraventricular systoles. 

### 3.2. Patient 2

The second patient is a 71-year-old woman with a history of hypothyroidism managed by replacement treatment, with a COVID-19 infection diagnosed on 6 March 2021. She has a secondary education. She was hospitalized previously due to high blood pressure and arrhythmia. She has never smoked. 

**Figure 2:**
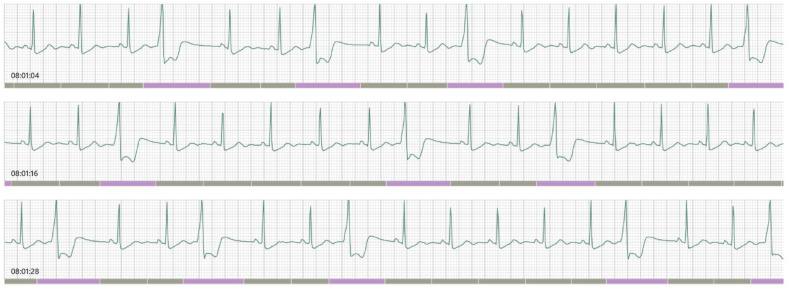
ECG reading.

The patient was treated on an outpatient basis, and reported non-specific pains in the chest, dyspnea, episodes of impaired consciousness and headaches. The chest X-ray in July 2021 showed both the heart and lungs within normal limits. The patient received an Aidmed recorder for a period from 13 August 2021 to 23 August 2021. In total, 9 h 3 min of measurements were recorded, during which time 11 episodes of elevated pulse (over 100 beats/min) were registered (Table 2). The ECG showed frequent disturbances, most probably of ventricular origin (Figure 2). Accurate diagnosis is not possible with a single-lead ECG; hence, on the first day of the study the patient was informed about the need for urgent cardiological consultation. 

The results of the patient’s questionnaires: the quality of sleep was evaluated as average. The patient pays attention to healthy nutrition. She does some light but regular physical exercise. On the EQ_5D_5L life quality scale, the patient indicated some slight trouble walking. She feels severe pain and discomfort. She is moderately anxious or depressed. She evaluated her health as average (50/100 points). In the WHOQOL_Bref life quality questionnaire, the patient indicates average or moderate physical, mental and social limitations in functioning, and evaluated her quality of life as good. She did not indicate any problems on the scale measuring the sense of loneliness (The UCLA LS). She did not find it difficult to operate the Aidmed system but needed to learn many things before she could begin working with the system. Despite having no previous contact with computers or smartphones, she was well guided by recruiters and telephone consultants.

Using a wireless pulse oximeter, included in the set, a patient measures saturation. During this study, many patients recorded lower values of blood oxygenation (drops by over 90%) and increased respiratory rates (over 20 breaths/min). Furthermore, the Aidmed device records such sleep disorders as episodes of sleep apnea, which call for further polysomnographic diagnosis. 

### 3.3. Patient 3

The third patient is a 71-year-old man, retired, with a history of arterial hypertension treated chronically with a beta-adrenolytic drug, and with first-degree obesity (BMI 30.07 kg/m^2^), a non-smoker for past 15 years (30 pack-years in the interview), who was diagnosed with SARS-CoV-19 infection on 5 December 2020. He has a secondary education and has not been hospitalized before. 

**Figure 3:**
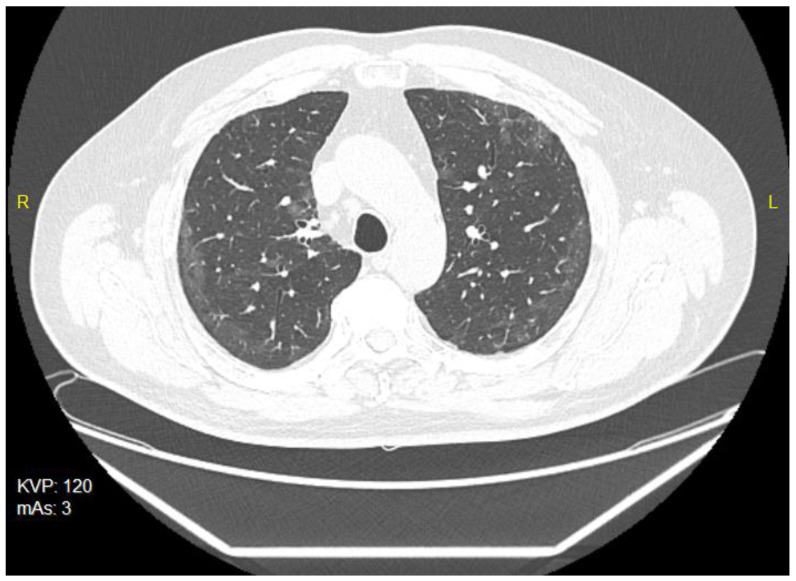
The chest CT scan of 5 February 2021—bilateral ground-glass opacities.

**Figure 4:**
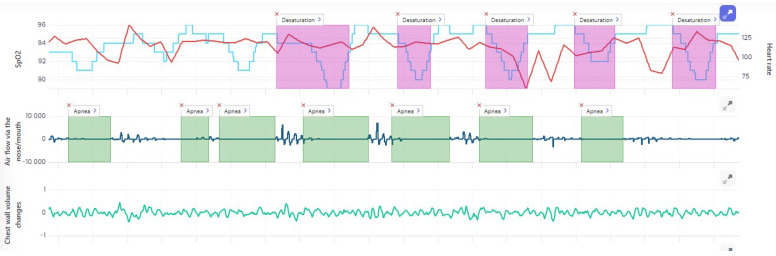
Sleep apnea reading, confirmed by polysomnography.

The patient was treated on an outpatient basis, as he complained of cough, breathlessness and fatigue. He was treated with azithromycin administered in a dose of 500 mg for 3 days. The chest X-rays revealed streaky parenchymal densities. The chest CT of 5 February 2021 showed areas of reduced transparency with mainly peripheral distribution, creating locally poorly saturated interstitial ground-glass opacities. There were also visible streaky parabronchial opacities—cicatricial—residual or post-inflammatory, especially at the base of lower lobes of both lungs, and traction of the nearby bronchi, showing slight signs of bronchiectasis (Figure 3). The Aidmed-assisted monitoring was conducted from 10 August 2021 to 23 August 2021. The total duration of the study was 31 h 38 min. The following events were recorded: 93 episodes of Tachypnea (over 25 breaths/min.), 242 episodes of decreased saturation (below 90%) and 716 episodes of tachycardia (over 100 beats/min.) (Table 3). Moreover, on 20 August 2021, during the night monitoring lasting 6 h 28 min, the patient experienced 194 episodes of sleep apnea, mainly in the supine position, and 4 episodes of shallow breathing (Figure 4). The average duration of an apneic episode was 24.02 s, while the longest one went on for 101 s. The AHI (number of apneic and dyspneic episodes) reached 29.98, which corresponds to moderately severe apnea. The lowest recorded blood oxygenation value was 89%, while the total time of saturation below 90% was 52 min. 

The patient was recommended further cardiological and pulmonological diagnosis. He underwent an overnight polysomnography on 20 October 2021—severe sleep apnea was diagnosed (AHI 70.8/h; ODI 75.4/h). Thus, the suspicion during AIDMED examination was confirmed.

The results of the patient’s questionnaires: the patient rated his quality of sleep as good; he does not pay attention to a healthy diet. He does some irregular physical exercise. He is not allergic, takes a dietary supplement regularly and occasionally consumes alcohol. On the EQ_5D_5L life-quality scale, the patient did not indicate any limitations and evaluated his health as good (80 points on a 100-point scale). In the WHOQOL_Bref life-quality questionnaire, the patient showed some small mental and social limitations in everyday functioning and evaluated the quality of his life as good. On the scale measuring the sense of loneliness (The UCLA LS), the patient did not show any significant problems except somewhat missing company. He experiences a low level of stress. The patient did not have any problems operating the Aidmed system. He did not admit to sensing any burden due to the pandemic and adhered to the restrictions.

## 4. Discussion

No guidelines for the treatment of patients with the post COVID syndrome have been issued so far. Patients who have recovered from SARS-CoV-2 infection can present various symptoms, such as fatigue, muscular pain, problems sleeping, cough, breathlessness, pains in the chest and heart palpitations. The Aidmed One recorder, presented in this research, allows one to verify some of these symptoms. 

The COVID-19 pandemic has stimulated the popularity of online service provision in medicine, as well as in other branches of the economy. Nevertheless, giving medical advice through telemedicine channels is certainly associated with a certain diagnostic risk. An assessment of a patient’s health through tele-advice may not be sufficiently accurate. A perfect example is the case reported in this paper of a 71-year-old woman and another one of a 46-year-old man. Both patients presented symptoms which were not directly connected with the so-called long-COVID, but were caused by the concomitant cardiac disorders. 

On the other hand, some of the symptoms reported by patients can be a consequence of long COVID. Table 1, Table 2 and Table 3 collate the data concerning the frequency of such symptoms as: cardiac arrhythmias, decreased saturation, Tachypnea or cough, presented by the patients. With respect to the 71-year-old woman, the symptoms she presented, including fatigue and faints, should rather be attributed to episodes of arrhythmia. The case of the 71-year-old man, in turn, is suggestive of the connection between the recorded episodes of tachycardia and persistent changes in the lung parenchyma indicative of interstitial disease. Such changes developing after COVID-19 infection could be one of the causes of respiratory disorders, depressed saturation, increased respiratory rate and, consequently, compensatory episodes of tachycardia. 

Natural disasters and epidemics pose many challenges in the provision of healthcare [18]. As a result, unique and innovative solutions are needed nowadays to respond adequately to critical needs of COVID-19 patients and others in need of healthcare. In order to prevent and restrict the transmission of COVID-19, patients and healthcare providers had to quite rapidly learn how to use telehealth models. Technological progress offers many possibilities in this regard [19]. The Aidmed One recorder is a device which makes it possible to monitor and make a comprehensive assessment of a patient’s health remotely. With this system, it is possible to make an objective evaluation of the general health status of a patient, including respiratory and cardiovascular disorders, for example arrhythmias (Figure 2). 

### Study Implications

Patients with long-COVID syndrome require comprehensive healthcare. It should relate to both the medical and psychological aspects. COVID-19 is a disease that mainly affects the respiratory system. Symptoms reported by patients, such as persistent shortness of breath, fatigue and weakness, may indicate multiorgan dysfunction. The assessment of oxygen saturation, respiration rate and its rhythm enable objective medical verification. Patients with low oxygen saturation, tachycardia, chronic cough or any abnormality in respiratory rhythm should be closely monitored and further diagnosed using available methods such as 12-lead ECG/echocardiogram, chest tomography or pulmonary functional tests such as spirometry or 6MWT. Mental-health problems are associated with mood swings, depression, anxiety, and sleep disorders. It is important to detect them using a battery of psychological and quality-of-life scales such as: EQ_5D_5L, WHOQOL_Bref, The UCLA LS, longitudinal symptom questionnaires, as well as PSS-10. These scales allow for quick mental-health screening. From a technical point of view, a remote, objective assessment of patients’ health, which can be conducted independently at home with machine learning algorithms producing alarms and helping with diagnostics, may save limited healthcare resources. Measurements of saturation, ECG and respiratory rate performed continuously for several dozen minutes per day are more reliable than the several-second recording of saturation carried out in a doctor’s office. They allow measurement of variability in the parameter values and have a holistic view of the patient.

Technologies used in telemedicine are useful and cost-effective methods employed to ensure access to high-quality health services and healthcare outcomes [20,21]. They also contribute to the lowering of healthcare costs [22]. However, it is obvious that telemedicine struggles with many problems. These include limitations in the use of modern technologies, patients’ inability to operate them and possible technical obstacles [23]. All these difficulties have been experienced in the study described. 

As early as the qualification stage, it was discovered that the type of mobile telephone owned by a patient could be problematic as regards the tested system. Many patients did not have a telephone with an operating system compatible with Aidmed, which would exclude them from the study. This problem was solved by providing these patients with a compatible mobile telephone together with an Aidmed device. 

Next, it was discovered that some patients needed to be retrained in the use of the device. Hence, all patients at all stages of the study had access to a technical consultant, who could support them in using the Aidmed One system correctly. It is worth emphasizing that, unlike popular lifestyle solutions available on the market, the provision of telemedicine services should be egalitarian and inclusive to the highest possible degree. Hence, digitally excluded patients, especially the elderly, should receive additional assistance. In this study, patients were also provided with an easy way to return the device at the termination of their monitoring, which proved to be extremely convenient, particularly for the individuals living away from the center where the study was performed. 

The provision of online or telephone consultations by a doctor, as well as the implementation of an adequate course of treatment without an actual appointment to see a doctor, is often met with patients’ reluctance, primarily because they are used to direct patient–doctor contact [24]. Protasio Lemos da Luz emphasized that face-to-face patient and doctor rapport is extremely important for making a correct assessment of a patient’s condition. This author reported a case of a patient with a tumor pressing on the trachea, which could only be diagnosed by direct physical examination of the patient [25]. However, the mentioned article had been published before the outbreak of the COVID-19 pandemic. While it is true that telemedicine will never replace traditional medical appointments, the application of modern technologies can improve them, just like social media and emails have facilitated traditional correspondence. 

## 5. Conclusions

During the epidemic, when contact with patients is limited, it is difficult to monitor the health of patients after they have been discharged from hospital. However, modern telemedicine solutions can make the process of diagnosing patients more efficient, while telemedicine can improve the patient–doctor rapport by enabling the doctor to make an objective assessment of a patient’s health remotely. This can significantly reduce the level of stress experienced by a patient due to a slow recovery from long COVID. Telehealth solutions also enable doctors to diagnose significant disturbances in how the patient’s body functions, which should undergo further diagnosis and treatment. 

The preliminary results of our study show that the application of advanced analysis with artificial intelligence components enables the detection of certain health complications, including ones connected with cases of long-COVID. The use of telemedicine solutions benefits both patients, who receive a better response to their health needs, and doctors, who gain deeper insight into their patients’ health problems. 

However, the implementation of telemedicine solutions calls for great flexibility and needs to engage multidisciplinary teams, who will respond and solve patients’ problems on an ongoing basis. 

### Limitation

This paper presents the preliminary results of an ongoing observational study, carried out by several research centers, in which patients’ health is monitored with the aid of remote devices. Undoubtedly, the results recorded with telemedicine devices must be interpreted by well-experienced medical personnel. In this study, it has been noted that an erroneously fitted respiratory canula could lead to a lack of signal recordings. Moreover, the ECG readings in obese patients can be falsified due to some disturbances of the signal transmitted from a one-lead ECG transducer caused by excess lipid tissue. Another example is dark nail varnish, which falsifies saturation readings. Furthermore, the results presented in this paper (e.g., ECG readings or measurements taken during sleep) need to be verified by complete diagnostic tests, e.g., with 12-lead ECG tests or full polysomnographic examination. It should be added that the results discussed in this paper originate from one center involved in the study, namely The Centre for Pulmonary Diseases in Olsztyn, Poland, and from a small population examined up to now (77 included patients). However, it was important for the authors of this paper to draw attention to the possibilities that telemedicine offers in the scope of monitoring and evaluating patients’ health. It is hoped that the calculated values of patients’ vital signs will help to determine reference standards in the future, and the tested system will be a valuable diagnostic instrument to assess a patient’s health at home, without hospitalization. 

## Figures and Tables

**Table 1:** Events recorded during the whole study period.

	Average Duration of An Event	Standard Deviation of Duration	Number of Events	Threshold	Number of Events/Minute
**Cough**	-	-	27	1	0.07
**Low SpO_2_**	93.49 s	1.51 s	38	<90	0.09
**High pulse**	96.88 s	6.98 s	191	>100	0.48
**Tachypnea**	15.06 s	3.95 s	2	>25	0.00

**Table 2:** Events recorded during the whole study period.

	Average Duration of An Event	Standard Deviation of Duration	Number of Events	Threshold	Number of Events/Minute
**Cough**	-	-	0	1	0.00
**Low SpO_2_**	95.15 s	1.50 s	6	<90	0.01
**High pulse**	73.40 s	11.06 s	11	>100	0.02
**Tachypnea**	19.25 s	4.60 s	31	>25	0.06

**Table 3:** Events recorded during the whole study period.

	Average Duration of an Event	Standard Deviation of Duration	Number of Events	Threshold	Number of Events/Minute
**Cough**	-	-	16	1	0.01
**Low SpO_2_**	94.03 s	1.71 s	242	<90	0.13
**High pulse**	81.48 s	24.31 s	716	>100	0.38
**Tachypnea**	19.71 s	3.85 s	93	>25	0.05

## Data Availability

The datasets used and/or analyzed during the current study are available from the corresponding author on reasonable request.

## Supplementary Materials

The following supporting information can be downloaded at: https://www.mdpi.com/article/10.3390/ijerph19095268/s1, Table S1: Advantages and disadvantages of the telemedicine during the COVID-19 pandemic based on previous studies; Figure S1: Patients flow for Long-COVID cohort of the study. References [26,27,28,29] are cited in the supplementary materials.
