# Supplementary material for: Telemonitoring in Long-COVID Patients—Preliminary Findings
Source: Int J Environ Res Public Health. 2022 Apr 26;19(9):5268. doi: 10.3390/ijerph19095268 (PMC9103243; doi:10.3390/ijerph19095268)
Supplement: Supplementary file 1 [file ijerph-19-05268-s001.zip › ijerph-1645343-supplementary.pdf]

Table S1. Advantages and disadvantages of the telemedicine during the COVID-19 pandemic based on previous studies.

1. Study	2. Advantages	3. Disadvantages
4. 26. Colbert, G.B.; Venegas-Vera, A.V.; Lerma, E.v. Utility of telemedicine in the COVID-19 era. Rev. Cardiovasc. Med. 2020, 21, 583–587. https://doi.org/10.31083/j.rcm.2020.04.188. [26]	- possibility of contact with a doctor and maintaining social distance, quarantine<br>- medical triage	- limitation of physical examination<br>- no access to imaging and laboratory tests
5. 27. Hincapié, M.A.; Gallego, J.C.; Gempeler, A.; Piñeros, J.A.; Nasner, D.; Escobar, M.F. Implementation and Usefulness of Tele-medicine During the COVID-19 Pandemic: A Scoping Review. J. Prim. Care Community Health 2020, 11, 2150132720980612. https://doi.org/10.1177/2150132720980612. [27]	- carrying out the "Forward triage"<br>- reducing the risk of infection<br>- minimizing the use of personal protective equipment	- inability to assess the patient's nonverbal communication
6. 28. De Albornoz, S.C.; Sia, K.-L.; Harris, A. The effectiveness of teleconsultations in primary care: Systematic review. Fam. Pract. 2022, 39, 168–182. https://doi.org/10.1093/fampra/cmab077. [28]	- - lower cost compared to a face to face visit	- socio-economic barrier<br>- more consultations
7. 29. Shaker, M.S.; Oppenheimer, J.; Grayson, M.; Stukus, D.; Hartog, N.; Hsieh, E.W.; Rider, N.; Dutmer, C.M.; Vander Leek, T.K.; Kim, H.; et al. COVID-19: Pandemic Contingency Planning for the Allergy and Immunology Clinic. J. Allergy Clin. Immunol. Pract. 2020, 8, 1477–1488.e5. https://doi.org/10.1016/j.jaip.2020.03.012. [29]	- reducing the exposure of healthcare professionals to potentially infected patients<br>- maintaining social distance<br>- quick assessment of patients for COVID-19 infection<br>- selection of patients' diagnoses and their severity	- traps of virtual care<br>- incorrect medical diagnoses

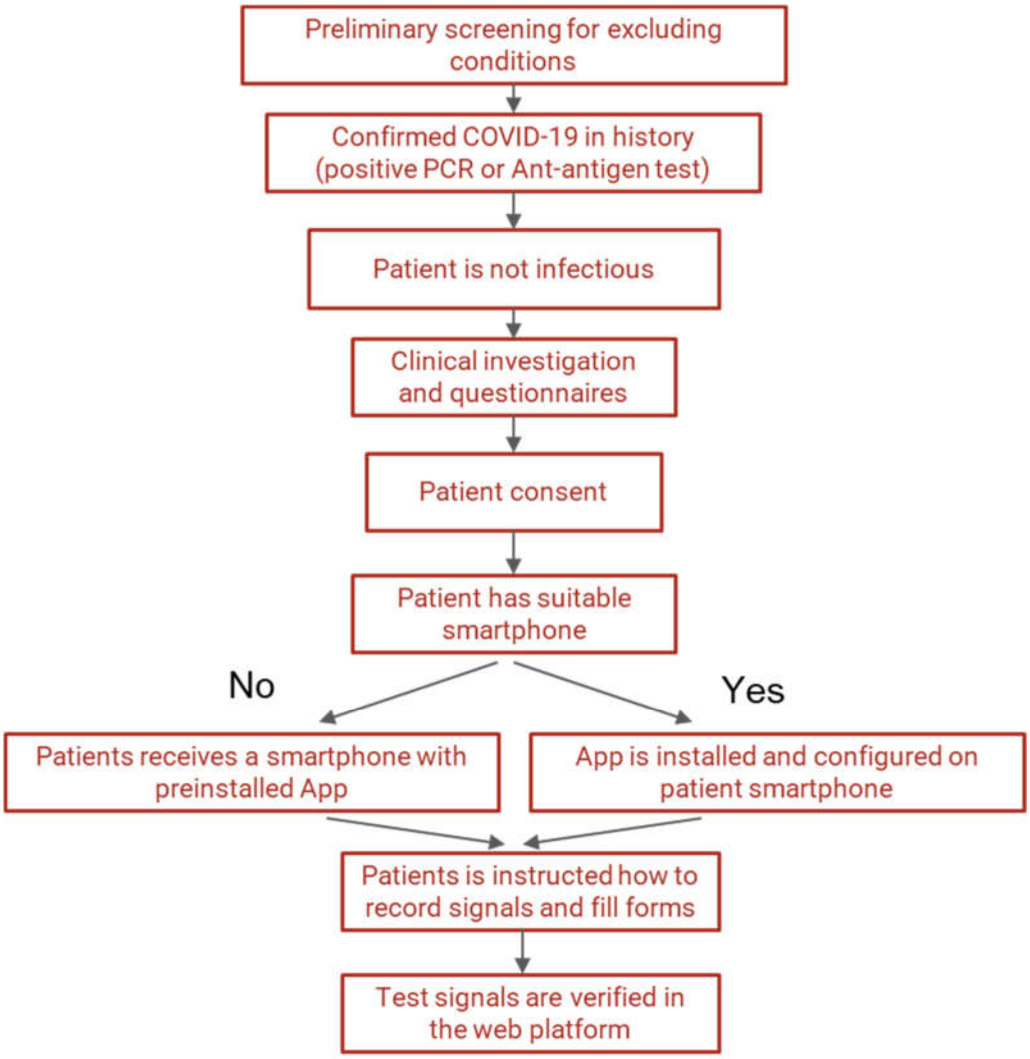

Figure S1: Patients flow for Long-COVID cohort of the study.
